# Distribution of fitness effects of cross-species transformation reveals potential for fast adaptive evolution

**DOI:** 10.1038/s41396-022-01325-5

**Published:** 2022-10-12

**Authors:** Isabel Rathmann, Mona Förster, Melih Yüksel, Lucas Horst, Gabriela Petrungaro, Tobias Bollenbach, Berenike Maier

**Affiliations:** 1 Institute for Biological Physics, University of Cologne, Zülpicherstr. 47a, 50674 Köln, Germany; 2 Center for Data and Simulation Science, University of Cologne, Köln, Germany; 3 Center for Molecular Medicine Cologne, University of Cologne, Köln, Germany

**Keywords:** Molecular evolution, Bacterial genetics

## Abstract

Bacterial transformation, a common mechanism of horizontal gene transfer, can speed up adaptive evolution. How its costs and benefits depend on the growth environment is poorly understood. Here, we characterize the distributions of fitness effects (DFE) of transformation in different conditions and test whether they predict in which condition transformation is beneficial. To determine the DFEs, we generate hybrid libraries between the recipient *Bacillus subtilis* and different donor species and measure the selection coefficient of each hybrid strain. In complex medium, the donor *Bacillus vallismortis* confers larger fitness effects than the more closely related donor *Bacillus spizizenii*. For both donors, the DFEs show strong effect beneficial transfers, indicating potential for fast adaptive evolution. While some transfers of *B. vallismortis* DNA show pleiotropic effects, various transfers are beneficial only under a single growth condition, indicating that the recipient can benefit from a variety of donor genes to adapt to varying growth conditions. We scrutinize the predictive value of the DFEs by laboratory evolution under different growth conditions and show that the DFEs correctly predict the condition at which transformation confers a benefit. We conclude that transformation has a strong potential for speeding up adaptation to varying environments by profiting from a gene pool shared between closely related species.

## Introduction

Horizontal gene transfer (HGT) can enhance the speed of bacterial adaptation to new environments and generate intra-species diversity [1–4]. The simplest mechanism of HGT is transformation. During transformation, bacteria take up DNA from the environment and integrate segments of the newly acquired DNA into their chromosomes by homologous recombination [5, 6]. While the mechanism of transformation is well characterized, there is an ongoing debate about costs and benefits of bacterial transformation as detailed in the following.

Benefits of transformation include the acquisition of novel functions like antibiotic resistance [7–9] or adaptation to novel carbon sources [10]. Transformation can also benefit bacteria by purging the genome from deleterious mutations and mobile genetic elements, or by recombining beneficial mutations that would compete in asexual populations [11–14]. On the other hand, transformation may introduce various costs including reduced RNA stability, protein activity, codon usage mismatch, or disruptive epistasis at the network level [15–17]. Various laboratory evolution experiments have addressed costs and benefits of transformation. These experiments were carried out under different experimental conditions and yielded a broad range of fitness effects of gene transfer [13, 18–22]. In an early study, Baltrus et al. found that the rate of adaptation of transformable *Helicobacter pylori* to a novel environment was higher than the rate of transformation-inhibited bacteria [13]. Other studies report that fitness effects of transformation were dependent on external stress [18] or growth phase [19]. We have studied the evolution of competent *Bacillus subtilis* in the presence of genomic DNA from *Bacillus spizizenii* (formerly *B. subtilis* subsp. *spizizenii*) [23]. Transformation benefitted *B. subtilis* during stationary phase and we found evidence for genome-wide positive and negative selection [23]. Therefore, it is important to understand and even predict conditions, where transformation by a specific donor benefits the recipient.

One central ingredient for predicting evolution is the distribution of fitness effects (DFE) [24]. It characterizes the spectrum of beneficial and deleterious genomic changes available to the evolving organism. This spectrum is usually represented by a library of strains with different specific genomic sequence modifications. So far, libraries containing strains with single mutations [25], multiple mutations [26, 27], and gene deletions [28] have been used to characterize the DFEs. The fitness effects were quantified either by measuring growth rates [25], by determining the selection coefficients in competition experiments [27, 29], indirectly by Bayesian inference [26], or at the single cell level [30]. In general, mutations or deletions occurring in the absence of selection shifted the DFE towards decreased fitness and only few mutations or deletions increased the fitness compared to their ancestors. In contrast, the DFE of transformation is poorly characterized. One study shows that inserting randomly chosen genes from different species into *Salmonella* chomosome caused no or mildly deleterious fitness effects [31]. Another study shows that insertion of different genes from *Salmonella typhimurium* into *E. coli* caused strong fitness costs that were dependent on the expression levels of the inserted genes [32]. The fitness effects of orthologous replacement have been investigated at the level of a single gene and shown to be mostly deleterious for different donor species [15, 33, 34]. To the best of our knowledge, the DFE of genome-wide transformation has not been characterized so far.

In this study, we characterized the DFE of cross-species transformation. *B. subtilis* was transformed by genomic DNA from *Bacillus vallismortis* to generate hybrid libraries. By competition between the hybrids and the recipient, we determined the DFE. In complex growth medium, we found strongly beneficial transfers that bear potential for rapid adaptation. In different growth environment, we found evidence for positive and negative synergistic pleiotropy as well as fitness trade-offs. Finally, we scrutinized the predictive value of our DFEs by laboratory evolution and found that the net fitness effects of transformation agree well with our predictions. Thus, our study is a significant step towards making the effects of transformation on bacterial evolution more predictable.

## Material and methods

### Strains and media

All experiments were performed with recipient strain Bs166, derived from *B. subtilis* BD630, and reporter strain Bs175, carrying an additional gene encoding GFP (RS). Donor strains are *B. spizizenii* NRRL B-14472/W23 (hybrid library BSPIZ) and *Bacillus vallismortis* DV1-F-3 (hybrid libraries BVAL, BVAL_single and evolved hybrid libraries). Experiments were performed in either complex medium (CM), defined medium (DM) or defined medium with glycerol as sole carbon source (DM_glycerol_).

We used whole genome sequencing data to detect orthologous recombinatios, insertions, deletions and duplications [23]. Reads are mapped using Burrows-Wheeler Aligner (v.0.7.17) [35], then processed with the mpileup function from samtools (samtools 1.8) and the variants are called with the call function from bcftools (bcftools 1.8) [36]. Detailed information to strains, media and sequencing data analysis can be found in Supplementary Methods.

### Generation of hybrid libraries

Random replacement hybrid libraries BVAL and BSPIZ were created by transforming recipient Bs166 cells with genomic DNA from *B. vallismortis* or *B. spizizenii*, respectively, and subsequently picking 88 hybrids from single colonies.

For the BVAL_single library, randomly picked single donor genes from *B. vallismortis* were replaced in the recipient entirely without selective markers [37] (details in Supplementary Methods, Fig. S1). BVAL_single consists of 24 strains each having a different gene fully replaced by the donor’s ortholog (Dataset S1) and additional 19 strains with a partial replacement.

#### Experimental evolution

An evolution experiment was performed with 88 hybrid populations created from one transformation step with *B. vallismortis* DNA. Populations were grown in exponential phase in parallel for ~450 generations in either CM or DM. Experiments ran for different periods of time as generation time depended on the growth medium (Table S3). After ~450 generations, one clone per population was picked and collected in the monoclonal hybrid libraries BVALevoCM and BVALevoDM. As a reference for both libraries, 88 wells of the recipient Bs166 were evolved and libraries RECevoCM and RECevoDM were generated. The evolution experiment was performed on an automated system integrated by the company HighRes Biosolutions. Detailed information available in Supplementary Methods.

#### Determination of selection coefficients

For each created library and the respective control, selection coefficients are measured and represented as distribution of fitness effects (DFE). Strains were competed against the reporter strain (RS) in different growth media and under different temperatures and growth conditions (Table S2). At the start *t*_0_ and end time point *t* of competition, fraction *x*_*i*_ of the strain of interest and *x*_*RS*_ of the reporter strain are determined with a flow cytometer. The selection coefficient was calculated as $s_{i,RS} = \frac{{t_g}}{{t - t_0}}ln\left( {\frac{{x_i\left( t \right)/x_{RS}\left( t \right)}}{{x_i\left( {t_0} \right)/x_{RS}\left( {t_0} \right)}}} \right)$, where t_g_ is the generation time of the recipient in the respective media (Table S3). For the DFEs, the selection coefficients *s*_*i,r*_ were calculated relative to the recipient’s fitness measured on the same experimental plate (Details to experimental procedure and analysis of flow cytometry data are explained in Supplementary Methods).

## Results

### Characterization of hybrid libraries formed between *B. subtilis* and *B. vallismortis*

In this study, *B. subtilis* served as a recipient species and the closely related *B. vallismortis* as a donor for gene transfer. *B. subtilis* and *B. vallismortis* share a core genome of 3.5 Mbp with an average sequence divergence of 7.4%. Additionally, *B. subtilis* and *B. vallismortis* both have an 0.7 Mbp accessory genome. By creating hybrids between these two species and determining the DFE, we ultimately aimed at understanding how bacterial transformation can drive adaptive evolution.

**Fig. 1:**
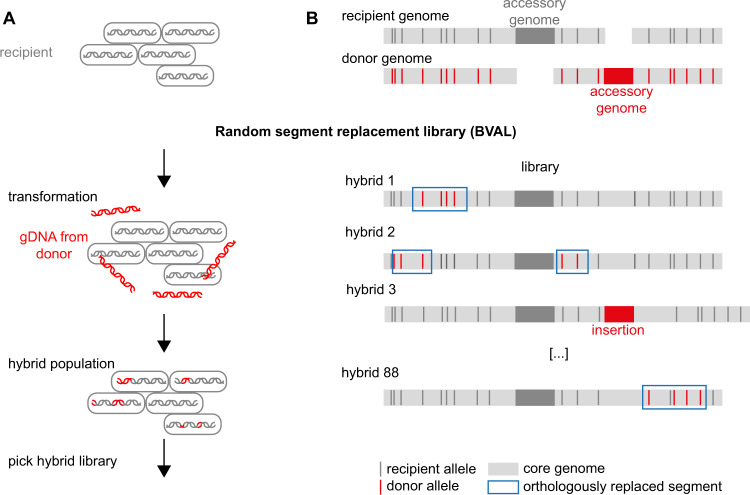
Library preparation. **A** The BVAL random segment replacement library was generated by transforming the recipient with genomic DNA of *B. vallismortis* for 2 h, plating, and picking of single colonies each consisting of a monoclonal hybrid. **B** The library consists of hybrids between donor and recipient. Random segments carry the donor alleles of core genes, deletions, and insertions from the donor’s accessory genome.

The hybrid library BVAL consists of 87 strains in which the recipient is genetically modified through transformation by genomic donor DNA (Fig. 1A). Between 0.01 and 0.66% of the core genome were orthologously replaced, i.e. a DNA segments belonging to the core genome of the recipient strain were replaced by a segment of the donor with similar sequence (Fig. 1B). On average, orthologous recombination replaced (0.1 ± 0.2)% of the core genome (including all sequenced strains) (Table S1) and affected 6 ± 11 genes at least partially. Besides orthologous replacements, deletions and de novo SNPs were detected. The library was designed to reflect the population of hybrids generated after 2 h of transformation by donor DNA and thus not all hybrid clones were genetically different from the recipient. In the hybrid library BVAL_single, random genes were orthologously replaced (Dataset S1, Fig. S1). By contrast to the BVAL library, intergenic regions were not affected by gene transfer in the BVAL_single library.

### Cross-species transfer has potential to enhance fitness

We determined the DFE of transformation in competence medium, a complex medium (CM) used for studying competence and transformation. We obtained the DFE by conducting competition experiments between each strain of the libraries and a fluorescent reporter of the recipient and measuring the selection coefficients as described in the Methods.

**Fig. 2:**
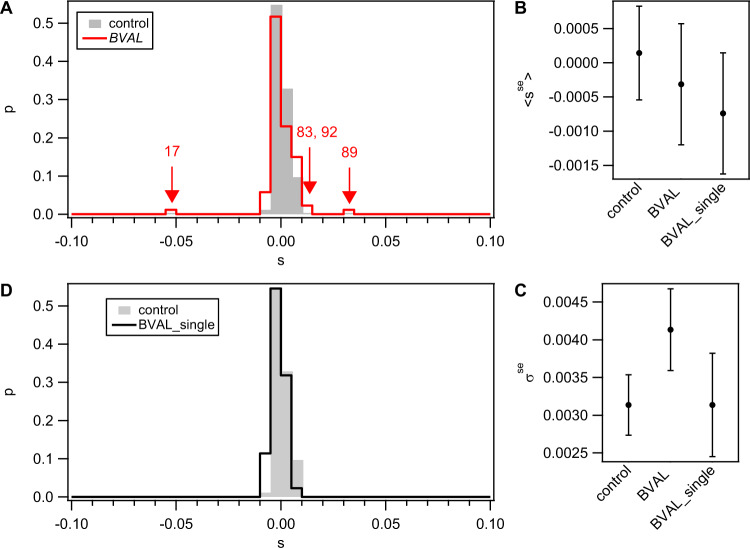
Distribution of fitness effects of BVAL and BVAL_single libraries. DFE was determined by competition against *gfp* reporter strain (Bs175) during exponential phase in complex medium for 4 h. Grey: Control DFE obtained from competition experiments between recipient (Bs166) and *gfp* reporter strain (grey). **A** Distribution of selection coefficients *s* of the BVAL library. Arrows denote outliers (large effect transfers) from the distribution derived at a significance level of *α* = 0.05 after Bonferroni correction. Numbers denote respective strains of BVAL. **B** Mean selection coefficients of core distributions excluding outliers <s^s^>. Error bars: confidence intervals obtained from bootstrap analysis. **C** Standard deviation of selection coefficients of core distributions excluding outliers *σ*^*se*^. Error bars: confidence intervals obtained from bootstrap analysis. **D** Distribution of selection coefficients *s* of the BVAL_single library.

All DFEs were be compared to a control DFE which characterizes the resolution of our setup. For the control DFE, we determined the selection coefficients of 82 independently growing recipients competing against their own reporter. The control distribution obtained is centered around *s*_*control*_ = 0.0001 ± 0.0007 (mean ± confidence interval) (Fig. 2). Using a Kolmogorov-Smirnov-test (KS-test), we find that the distribution is consistent with a normal distribution. The standard deviation is *σ*_*control*_ = 0.0031 ± 0.0004.

We measured the DFE of the BVAL library (Fig. 2A). By resampling we show that our sample size adequately captures the global shape of the underlying DFE (Fig. S2). Three strains had strongly positive selection coefficients and one strain had a strongly negative selection coefficient. We defined outliers from the control distribution, i.e. strains with large fitness effects, by a significance level of *α* = 0.05. To account for multiple testing, the Bonferroni correction [38] was applied. Using this criterion, we find three positive and one negative outlier. Henceforth, “large effect transfers” will denote transfers that cause these outliers.

The central portion of the distribution appeared to be broader than the control distribution (Fig. 2A). This could indicate “small effect transfers”, whereby individual strains would not show significant fitness effects but the distribution as a whole would still differ from the control. To analyze this, we obtained the core distribution by removing the large effect transfer outliers, which left us with a DFE dominated only by small effects (se), DFE^se^. We find that the mean of the DFE^se^ is comparable to the control (Fig. 2B). By contrast, the DFE^se^ of BVAL shows a significantly larger standard deviation (Fig. 2C), indicating the existence of multiple small effect transfers with positive and negative fitness effects. An individual strain with a small effect transfer is not significantly different from the control distribution, but DFE^se^ as a whole is significantly different. This result was also confirmed by a Bartlett test.

The DFE of the BVAL_single library showed a small shift to negative selection coefficients but no outliers from the control distribution (Fig. 2D). Neither the mean selection coefficient nor the standard deviation was significantly different from the control distribution (Fig. 2B, C). When strains with full gene replacements and partial replacements were analyzed separately, no significant difference was found (Fig. S3). Taken together our data do not reveal strong fitness effects of single gene replacement. We conclude that in complex medium the DFE of transformation shows large effect and small effect transfers. In particular, 3 out of 88 transformants have strongly enhanced fitness.

### Transfers from more closely related donor species create higher genomic variability but weaker fitness effects

Different donors are expected to generate different DFEs of transformation. Here, we investigate the DFE of transformation using *B. spizizenii* as donor. *B. subtilis* and *B. spizizenii* share a core genome of 3.6 Mbp with an average sequence divergence of 6.8 %. The size of the core genome is comparable to *B. vallismortis*, but the sequence divergence is lower. Additionally, *B. subtilis* has 0.6 Mbp accessory genome and *B. spizizenii* has 0.4 Mbp. The library BSPIZ was generated using the same method as for BVAL. The mean fraction of orthologous replacement was (0.5 ± 0.8) % (Table S1). On average, 21 ± 31 genes were hit by orthologous replacement. Next to orthologous replacement, insertions from the accessory genome of the donor and deletions were detected. In total, the strains of BSPIZ showed more gene transfer compared to BVAL.

**Fig. 3:**
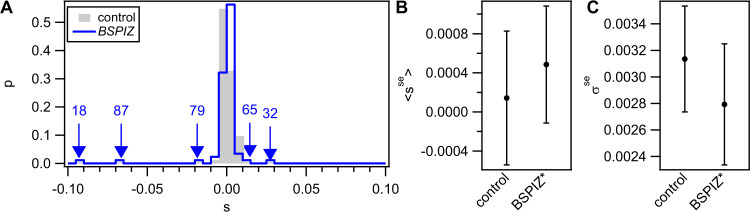
Distribution of fitness effects of BSPIZ library. DFE was determined by competition against *gfp* reporter strain (Bs175) during exponential phase in complex medium for 4 h. Grey: Control DFE obtained from competition experiments between recipient (Bs166) and *gfp* reporter strain (grey). **A** Distribution of selection coefficients *s*. Arrows denote outliers (large effect transfers) from the distribution derived at a significance level of *α* = 0.05 after Bonferroni correction. Numbers denote respective strains of BSPIZ. **B** Mean selection coefficients of core distributions excluding outliers <s^se^>. Error bars: confidence intervals obtained from bootstrap analysis. **C** Standard deviation of selection coefficients of core distributions excluding outliers *σ*^*se*^. Error bars: confidence intervals obtained from bootstrap analysis.

We measured the DFE of the BSPIZ library in complex medium (Fig. 3A). Two strains were characterized as positive outliers and three strains as negative outliers. After removing these large effect transfer outliers, we investigated whether the core distribution was influenced detectably by small effect transfers. We found that neither the mean selection coefficient of the core distribution nor the standard deviation was significantly different from the control (Fig. 3B, C). In summary, a more closely related donor generated more gene transfer. The DFE showed no detectable small effect transfers, but multiple beneficial and deleterious strong effect transfers.

### Different growth conditions strongly affect the DFE showing different types of pleiotropy

The heterogeneity generated by transformation likely enables adaptation to fluctuating environments. We expect different hybrids to show fitness effects under different experimental and environmental conditions. To find out how growth conditions affect the DFE, we measured the selection coefficients of the BVAL hybrids against the recipient under different conditions (Table S2). First, we assessed the influence of different growth conditions in complex medium. In particular, we addressed the effects of the lag phase and of increased temperature. Second, we maintained the original growth conditions but varied the carbon sources and amino acid compositions.

**Fig. 4:**
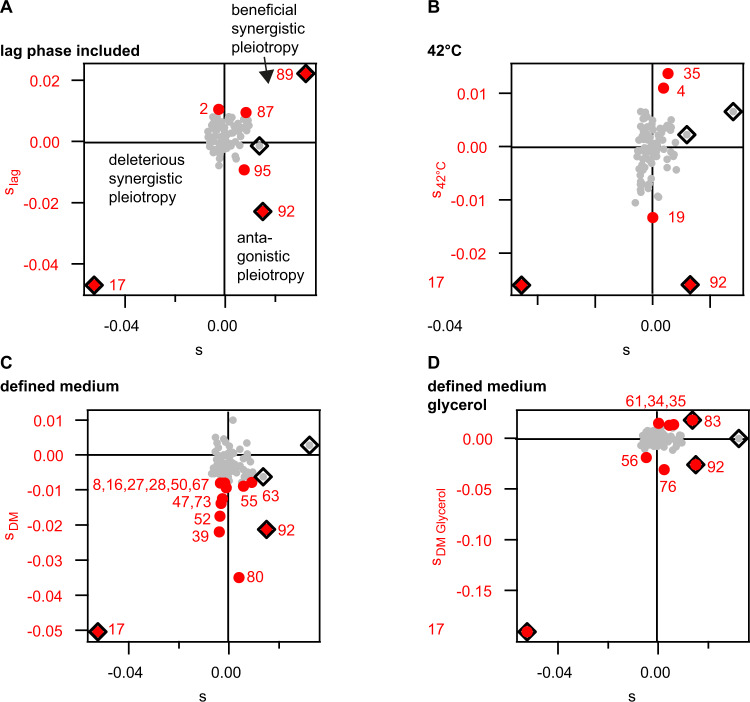
Fitness effects depend on growth conditions. Scatter plots of selection coefficients of BVAL determined under different growth conditions plotted against the selection coefficients in complex medium at 37 °C °C as shown in Fig. 2A. Red circles: outliers from DFE under different conditions, black diamonds: outliers from DFE in complex medium at 37 °C, grey: strains with small effect transfers. **A** Complex medium including the lag phase at 37 °C. **B** Complex medium excluding the lag phase at 42 °C. **C** Defined medium excluding lag phase at 37 °C. **D** Defined medium with glycerol as only carbon source excluding lag phase at 37 °C.

The lag phase introduces fitness effects that are distinct from changes in growth rate during exponential phase. The duration of the lag phase is a selective trait, for example in the presence of antibiotics [39]. In our experimental setup, fast escape from the lag phase will be detected as a benefit. To assess the fitness effects of the lag phase, we compared the selection coefficients of BVAL determined excluding (Fig. 2) and including (Fig. 4A) the lag phase. To include the lag phase, the competitors were harvested from stationary phase, and mixed with the reporter immediately. When the lag phase was excluded, the competitors were harvested from stationary phase, grown to exponential phase, and subsequently mixed. When we included the lag phase, we find six strong effect transfers (Fig. 4A, Fig. S4A) of which three are positive outliers and three are negative outliers. Using a scatter plot, we investigate correlations between conditions with and without lag phase (Fig. 4A). We find different types of pleiotropy between both conditions. Regarding outliers only, one strain (BVAL_89) shows beneficial synergistic pleiotropy, i.e. fitness is strongly increased under both conditions. Another strain (BVAL_17) shows deleterious synergistic pleiotropy. One strain (BVAL_92) shows antagonistic pleiotropy, suggesting that a trait has been transferred that confers a benefit during exponential growth in complex medium but a cost during escape from the lag phase. Several strains were defined as fitness outliers only under one of both conditions. To evaluate whether transfers with small fitness effects were prominent, we analysed the $DFE_{lag}^{se}$ after removing the outliers. We found that the mean selection coefficient was positive (Fig. S4B) and both the mean and standard deviation were significantly higher than in the control (Fig. S4C).

*B. vallismortis* has been isolated from Death Valley soil and it is conceivable that it is well adapted to higher temperatures. To investigate whether the hybrids benefit from the donor’s trait, we performed the competition experiment (excluding the lag phase) at a temperature of 42 °C. At this temperature, the generation time of the recipient was decreased slightly to (14.9 ± 0.2) min. Five strong effect transfers were detected; two positive outliers and three negative outliers (Fig. 4B, Fig. S4D). The positive outliers (BVAL_4, 35) are different from the positive outliers under the previously studied conditions, showing that different horizontally acquired genes confer a benefit in different environments. Again, BVAL_17 shows deleterious synergistic pleiotropy and BVAL_92 shows antagonistic pleiotropy (Fig. 4B). After removing the outliers, the standard deviation was significantly higher than the control standard deviation (Fig. S4F).

We investigated effects of different carbon sources on the DFE. First, we used a chemically defined medium (DM) where glucose, glutamate, and citrate were used as carbon source. The generation time in DM was increased to (39.0 ± 0.2) min. In DM, the DFE was shifted towards negative selection coefficients (Fig. 4C, Fig. S5A). We found 15 strong effect transfers and all of them were outliers towards negative fitness (Fig. S5A). Without outliers, the mean selection coefficient was negative with $< s_{DM}^{se} > = \left( { - 0.0026 \pm 0.0008} \right)$ (Fig. S5B). Thus, exponential growth at 37 °C in defined medium is the only condition under which we see a significant decrease in mean selection coefficients.

We characterized the fitness effects in defined medium with glycerol as the only carbon source (DM_Glycerol_). The generation time in DM_Glycerol_ was (48.3 ± 0.3) min. We found eight strong effect transfers, four of them (BVAL_34, _35, _61, _83) having a positive fitness effect (Fig. 4D, Fig. S5D). Compared to competition in complex medium, BVAL_83 showed positive synergistic pleiotropy.

In summary, the DFEs revealed beneficial transfers from *B. vallismortis* to *B. subtilis* under most but not all conditions studied. When comparing fitness effects of transformation under different conditions, we found evidence for synergistic and antagonistic pleiotropy. Several transfers were beneficial only under a single condition.

### Transformation confers a benefit in complex medium but not in defined medium

**Fig. 5:**
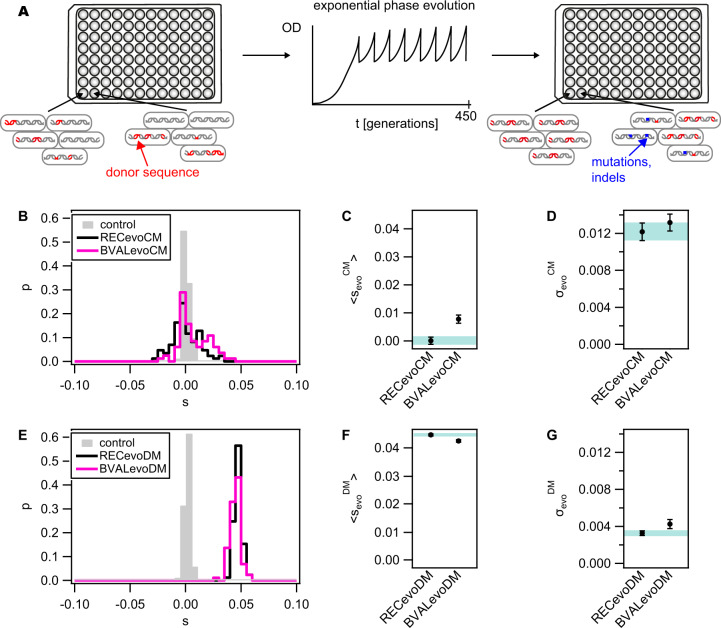
Fitness effects of transformation in evolving populations. **A** Sketch of the evolution experiment. 88 batches of the recipient (REC, Bs166) were transformed with donor DNA prior to the evolution experiment, creating a population consisting of different hybrids within a single batch. During laboratory evolution, the OD was monitored, and the batches were diluted repeatedly to maintain exponential growth. After ~450 generations (5 days in CM and 12.5 days in DM), a single clone was picked from each batch, creating a library of evolved strains (BVALevoCM in CM and BVALevoDM in DM). The same procedure was repeated using the untransformed recipient, creating RECevoCM and RECevoDM. The selection coefficients of the libraries were determined by competition against the reporter Bs175. **B**, **E** Distribution of selection coefficients in **B** complex medium and **E** defined medium. Grey: control (Bs166) without evolution, black: evolved recipient strains, pink: evolved hybrid strains. **C**, **F** Mean selection coefficients and **D**, **G** standard deviation in **C**, **D** complex medium and **F**, **G** defined medium. Error bars: confidence intervals obtained from bootstrap analysis. Shaded area: confidence level of evolved recipient data.

Reviewing the measured DFEs of gene transfer from *B. vallismortis* to *B. subtilis*, we identified two conditions that allow for a prediction on the course of adaptive evolution. First, we note that in complex medium, we found large effect beneficial transfers. Opposed to this, in defined medium, the mean selection coefficient was shifted to a negative value and no large effect beneficial transfers were found. Using this information, we predicted that bacteria benefit more from transformation in complex medium but less so in defined medium. To scrutinize this prediction, we designed a laboratory evolution experiment (Fig. 5A) that ran in both growth media. First, the recipient *B. subtilis* was transformed by gDNA from the donor *B. vallismortis* (Fig. 1A). For both CM and DM, the freshly generated hybrids were split into 88 wells. Thus, at the beginning of the evolution experiment, we had 88 populations each containing ~10^5^ different hybrid clones. For each condition, these populations evolved independently by growing exponentially for ~450 generations (Fig. 5A), i.e. 5 days in CM and 12.5 days in DM. The same experiment was performed with the untransformed recipient *B. subtilis* so that we could compare hybrid populations to populations without prior transformation. After ~450 generations, we assessed how the fitness in CM and DM had changed during evolution. To this end, we generated the evolved strain libraries by picking a random clone from each of the 88 populations. The evolved hybrids were represented by the libraries BVALevoCM and BVALevoDM for evolution in complex and defined medium, respectively. The distribution of selection coefficients relative to the recipient was determined using competition experiments.

In complex medium (CM), the mean selection coefficient of the hybrid library BVALevoCM $< s_{BVALevo}^{CM} > = \left( {0.008 \pm 0.001} \right)$ was significantly higher than the mean selection coefficient of the recipient library RECevoCM $< s_{RECevo}^{CM} > = \left( {0.000 \pm 0.001} \right)$ (Fig. 5B, C). This result shows that the transformation caused a fitness increase in CM as predicted by the DFE of BVAL. A KS-test shows that the distributions of selection coefficients of BVALevoCM and RECevoCM are significantly different (p = 0.003). The distributions of selection coefficients showed comparable broadening relative to the control distribution (Fig. 5B, D).

In defined medium the mean selection coefficient of the untransformed library RECevoDM $< s_{RECevo}^{DM} > = \left( {0.0447 \pm 0.0004} \right)$ was slightly but significantly higher than the selection coefficient of the hybrid library BVALevoDM with $< s_{BVALevo}^{DM} > = \left( {0.0425 \pm 0.0004} \right)$ (Fig. 5E, F). This result is consistent with the prediction based on the DFE of BVAL that transformation is not beneficial in defined medium. While the standard deviation of selection coefficient of RECevoDM was not significantly different from the standard deviation of the control distribution (Fig. 5G, Fig. 2C), the standard deviation of BVALevoDM was slightly increased (Fig. 5G). A KS-test shows that the distributions of selection coefficients of BVALevoDM and RECevoDM are significantly different (p = 0.005). We conclude that transformation by DNA from *B. vallismortis* confers a net benefit in complex medium, but introduces a cost in defined medium as predicted by the respective DFEs.

### Genetic variability is higher for bacteria evolved in complex medium than in defined medium

**Fig. 6:**
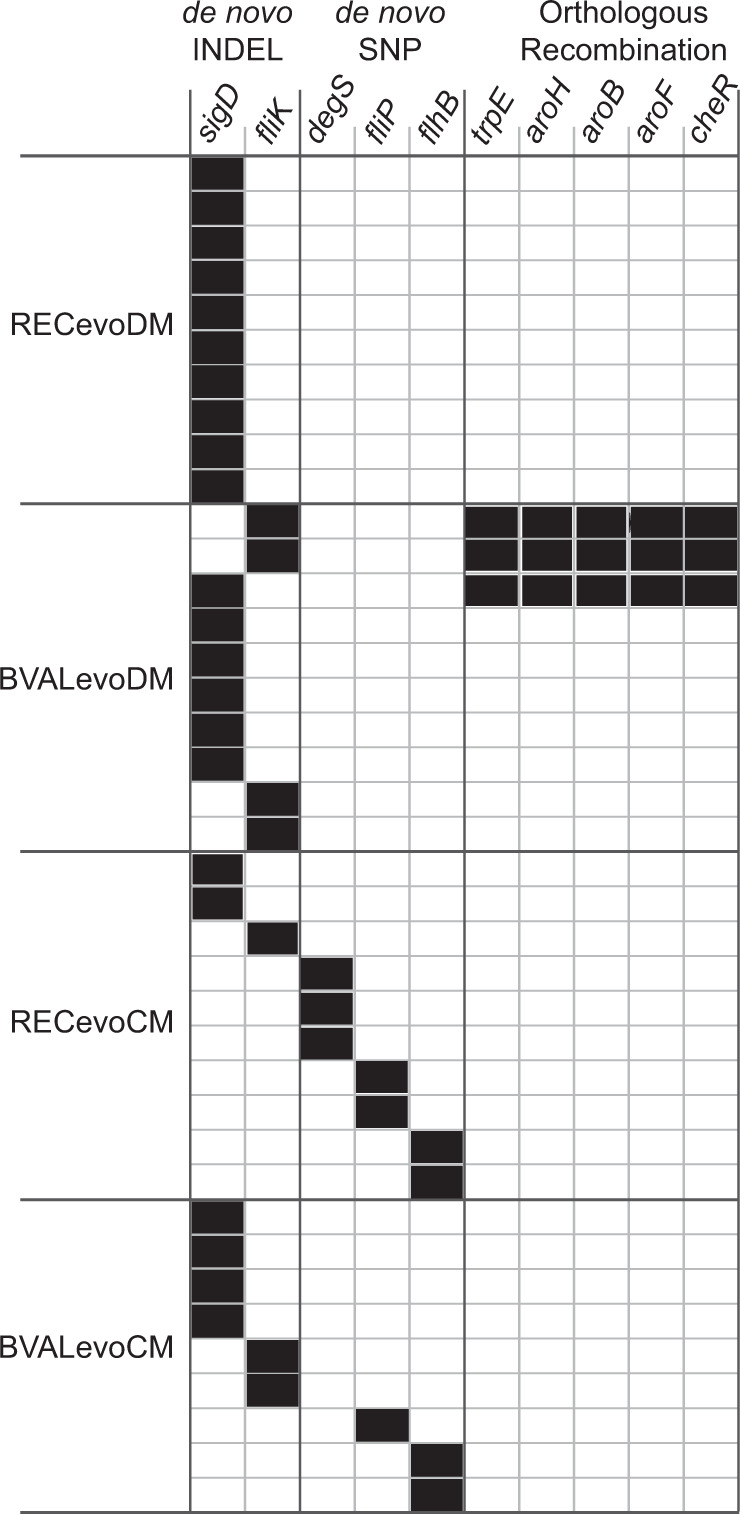
Hotspots of mutations, indels, and orthologous recombination. A hotspot was defined as a gene that showed sequence changes in at least two of the evolved strains. De novo INDEL: small insertion or deletion of bases within the gene, de novo SNP: single nucleotide polymorphism in the gene that does not arise from orthologous replacement with a donor segment, Recombination: recipient gene replaced by donor gene through orthologous recombination.

We assessed repeatability of orthologous recombination and de novo mutations. During the evolution experiment, de novo mutations occur and selection leads to their fixation. For the evolved recipients, this is the only source of genetic variation. For the evolved hybrids on the other hand, mutations occur against the background of the orthologous recombination. The distributions of selection coefficients in Fig. 5C, F suggested high repeatability in defined medium and lower repeatability in complex medium. To assess repeatability, we performed whole genome sequencing on the ten fittest hybrids of each evolved library (Dataset S2). We detected orthologous recombination as well as de novo mutations, including de novo indels and de novo SNPs. Here, we report genes that genetically changed in at least two strains as hotspots (Fig. 6).

In defined medium, we found that both evolved libraries, BVALevoDM and RECevoDM, had considerably increased fitness (Fig. 5E–G). This suggests that the better part of the fitness increase occurred independently of transformation and was caused by a few shared de novo mutations. Indeed, we found that all of the sequenced strains of RECevoDM had frame-shift mutations in the *sigD* gene encoding for an alternative sigma factor involved in flagella synthesis (Fig. 6) [40, 41]. The indels occurred within a poly-A stretch (Dataset S2) suggesting that *sigD* is phase-variable. No further hotspots were found for RECevoDM. Out of the ten strains sequenced for BVALevoDM (hybrids evolved in defined medium), six showed an indel in *sigD* and four had an indel in *fliK* encoding for a protein involved in controlling the hook length of the flagellum [42]. No further mutational hotspots were found. In addition to the de novo mutations, we found a recombination hotspot. In three out of ten strains, the operon *cheR*-*aroF*-*aroB*-*aroH*, and the adjacent gene *trpE* were affected by orthologous recombination.

For complex medium, we found a broader distribution of selection coefficients of BVALevoCM and RECevoCM (Fig. 5B–D), suggesting that evolution is less repeatable. In accordance with this, we found a higher diversity of hotspots in the sequenced strains. Nevertheless, all of the sequenced strains show indels or SNPs in flagella-related genes (Fig. 6). These include indels in *sigD* and *fliK*, and additionally, SNPs in genes responsible for forming the basal complex of flagella, *fliP*, and *flhB* as well as the two-component sensor kinase *degS* which affects the *sigD* regulon [43]. Even though orthologous recombination took place, we did not detect a recombination hotspot. Instead, many different genes were affected by recombination.

In summary, we found that repeatability of evolution is higher in defined medium than in complex medium consistent with the high selection coefficients in this medium.

## Discussion

In this work, we systematically address the effects of cross-species transformation on bacterial fitness. We show how its benefit depends on the growth context and use the DFEs to make predictions about environments that favour transformation during adaptive evolution. Results obtained by laboratory evolution support the predictive value of the DFEs.

We found qualitative differences between the DFEs of transformation and the previously characterized DFEs of single mutations and single gene deletions show qualitative differences [24–27]. For the latter, the centres of the DFEs were shifted towards deleterious fitness effects with few exceptions, including a mutator strain with a specific mutation spectrum [25]. Few mutations or deletions had positive fitness effects. For orthologous replacement investigated in this study, we expected that fitness effects were deleterious on average as a consequence of hybrid incompatibilities. Disruptive epistasis at the level of functional networks [23] and suboptimal gene expression levels [15, 33, 44] were likely to reduce the fitness of the hybrid strains. Here, we found little support for this expectation with one exception discussed below. Using two different donor strains and four different growth conditions, our data indicate that transformation by DNA from closely related species is fitness neutral. The DFE has a core distribution around s = 0 that tends to broaden as a result of gene transfer. For all tested conditions 2–5% of the hybrid strains had selection coefficients significantly higher than the control distribution, i.e. large effect beneficial transfers. 1–5% of the hybrid strains showed large effect deleterious transfers. The large effect beneficial transfers have potential for increasing the speed of adaptation. This suggests that *B. subtilis* can use a shared gene pool with closely related species to adapt to a variety of new environments. We found one interesting exception from the general trend described so far. In defined medium (DM), we identified fifteen large effect deleterious transfers and the core distribution was shifted to a negative value. We conclude that gene transfer from *B. vallismortis* to *B. subtilis* tends to confer a net fitness cost in DM. Currently, we can only speculate why the DFE in defined medium is qualitatively different from all other conditions probed in this study. The difference between complex medium and defined medium could be explained by the complexity hypothesis [17]; most likely, bacteria adapt “add-on” functions that work independently of strongly interconnected networks belonging to the central metabolism. In defined medium, bacteria metabolize glucose, glutamate, and citrate. The involved pathways are likely to be similar between the species and, therefore, orthologous replacements have small fitness effects. In complex environments, different alternative and poorly linked pathways enable bacteria to adapt to different growth environments. Therefore, genetic exchange in complex environment can confer a higher fitness advantage than in environments of low complexity. However, the DFE_glycerol_ in defined medium with glycerol as the only carbon source does not support this explanation, since we found strong effect beneficial transfers.

Based on the results obtained from the DFEs, we predicted that transformation conveys a fitness benefit in CM but less so in DM. An evolution experiment supported this prediction. Additionally, we found that repeatability of fitness effects, de novo mutations, and orthologous recombination was higher in defined medium. In DM, all sequenced strains carry an indel in *sigD* or *fliK* disrupting stable flagella formation. The latter gene plays an important role during the formation of the flagellum for robustly activating *sigD*, and we conclude that its frame-shift mutation confers the same functional change as inactivation of *sigD*. Inactivation of *sigD* has been shown to confer a benefit previously [45]. Even though we sequenced only one clone of each population, it is most likely, that this indel became fixed and conferred the fitness increase. This also explains why we see no strain with negative fitness effect, as evolution has most likely led to fixation in most populations. For the hybrid strains evolved in defined medium, we also find a hotspot of gene transfer encompassing the *cheR*-*aroF*-*aroB*-*aroH* operon, and the adjacent gene *trpE*. These genes are involved in the shikimate pathway responsible for synthesis of tryptophan, phenylalanine and tyrosine [46]. Defined medium does not contain these amino acids, and the transfer may enhance their production rate. While in DM transformation caused a cost at the genome-wide scale, this specific orthologous recombination was most likely selected for.

In complex medium, beneficial evolved hybrids have a higher genetic variety. Again, all of the mutational hotspots occurred within flagella-related genes. However, the diversity of flagella-related genes mutated in complex medium is higher than in defined medium. Unexpectedly, the untransformed recipient showed no net fitness increase in complex medium after ~450 generations. We suggest that the recipient is already well-adapted to the complex medium prior to the evolution experiment and mutations do not likely convey large benefits but rather broaden the DFE. Despite the net benefit of gene transfer, we found no hotspot of recombination in complex medium. We argue that this is due to the fact that the initial hybrid populations likely contain multiple different beneficial transfers. Each hybrid population initially comprises about 10^5^ cells and we have even observed beneficial transfers in the BVAL library made up by only 87 strains. Additionally, we hypothesize that fitness differences between beneficial transfers are smaller in complex medium and that this slows down fixation time. Consequently, beneficial hybrids might not yet have reached fixation in the population. For both the recipient and the control, we found multiple strains that even show decreased fitness. We suggest that complex medium sets the stage for more complicated population dynamics in which fixation is delayed and even hybrids with negative fitness effects can persist. It is also conceivable that interactions between different clones of one population increase the population fitness. By picking a single clone from each evolved population, we did not account for this possibility. Future experiments will have to address within population diversity as a function of time.

Here, we have designed the hybrid libraries to reflect fitness effects after one single cycle of transformation in *B. subtilis*. *B. subtilis* switches stochastically into the state of competence at high cell density and remains competent for ≈2 h [6, 47]. Therefore, our libraries were generated by transforming with donor DNA for this period of time. We show that within one cycle, different donor species create different degrees of genetic variation. The fraction of replaced genome is considerably (>2 fold) higher with *B. spizizenii* than with *B. vallismortis*, yet the fitness effects are stronger with *B. vallismortis*. While both DFEs contain strongly beneficial outliers, the DFE of *B. vallismortis* reveals additional small effect transfers, suggesting that fitness effects increase with increasing sequence divergence between donor and recipient. The mean lengths of the replaced segments were considerably shorter in BVAL (1.3 kbp) compared to BSPIZ (4.0 kbp). Whereas the mean segment length of the BVAL strains hardly exceeds the mean length of a single gene, the mean segment length of the BSPIZ strains encompasses multiple average-sized genes. Therefore, the probability that operons including their promoters are fully replaced is higher and the probability of disruptive epistasis of functional networks is lower, suggesting that the fitness effects of gene transfer from *B. spizizenii* are lower.

In this work, cells are generally kept in exponential growth phase during evolution and competition experiments. In this well-defined and reproducible condition, the number of cells increases rapidly compared to other growth phases and selection mainly acts on fast growth. Thus, restricting the experiments to this single phase facilitates mapping of genetic variations to their fitness effects. We show that adding another growth phase, such as the lag phase, to the growth condition has an impact on the hybrid fitness. We expect the same to be true if including the stationary growth phase, especially for *B. subtilis* where processes like cannibalism, sporulation, and biofilm formation will potentially have an impact on fitness effects [48]. We envision that our method can be used in the future to systematically assess the DFEs in different growth phases.

In conclusion, the DFE of transformation is systematically different from the DFE of mutations investigated previously. By contrast to the latter, there is no net shift to reduced fitness in most growth conditions. This difference may be explained by the fact that the exchanged sequences were functional in the donor. Our study corroborates the idea that a shared gene-pool between closely related species enables rapid adaptation to changing environments. In support of this idea, recent work showed that *B. subtilis* enters the state of competence more frequently if a closely related species is present [8]. In future studies, it will be interesting to find out how the prolonged presence of other species affects the fitness and genome dynamics of the recipient. In particular, it is currently unclear whether fitness effects of multiple transfers are additive or whether epistatic effects dominate. In terms of application, the predictive value of the DFEs will most likely become useful for predicting effects of transformation on the speed of antibiotic resistance evolution.

## Supplementary information

Supplemental Material

Dataset S1

Dataset S2

## Data Availability

The datasets generated during and/or analysed during the current study are available as Supplementary data and at NCBI SRA (BioProject PRJNA877563).
