# Correction to: Distribution of fitness effects of cross-species transformation reveals potential for fast adaptive evolution

**DOI:** 10.1038/s41396-023-01440-x

**Published:** 2023-05-31

**Authors:** Isabel Rathmann, Mona Förster, Melih Yüksel, Lucas Horst, Gabriela Petrungaro, Tobias Bollenbach, Berenike Maier

**Affiliations:** 1 Institute for Biological Physics, University of Cologne, Zülpicherstr. 47a, 50674 Köln, Germany; 2 Center for Data and Simulation Science, University of Cologne, Köln, Germany; 3 Center for Molecular Medicine Cologne, University of Cologne, Köln, Germany

**Keywords:** Molecular evolution, Bacterial genetics

Correction to: *The ISME Journal* 10.1038/s41396-022-01325-5, published online 12 October 2022

In the sentence beginning ‘One study shows that...’ in this article, the term ‘Escherichia coli’ should have read ‘Salmonella chomosome’

In this article ref. 31 was incorrect and should have been 31. Knöppel A, Lind PA, Lustig U, Näsvall J, Andersson DI. Minor fitness costs in an experimental model of horizontal gene transfer in bacteria. Mol. Biol. Evol.; 2014 31(5):1220–7; 10.1093/molbev/msu076

The original article has been corrected.

