# Supplementary material for: Distribution of fitness effects of cross-species transformation reveals potential for fast adaptive evolution
Source: ISME J. 2022 Oct 12;17(1):130–9. doi: 10.1038/s41396-022-01325-5 (PMC9751276; doi:10.1038/s41396-022-01325-5)
Supplement: Supplementary file 1 — Supplemental Material [file 41396_2022_1325_MOESM1_ESM.pdf]

**Supplementary information for**

# Distribution of fitness effects of cross-species transformation reveals potential for fast adaptive evolution

Isabel Rathmann*[1], Mona Förster*[1], Melih Yüksel[1], Lucas Horst[1], Gabriela Petrungaro[1], Tobias Bollenbach[1,2], Berenike Maier[1,3]

*contributed equally

[1] Institute for Biological Physics, Zülpicherstr. 47a, 50674 Köln, University of Cologne

[2] Center for Data and Simulation Science, University of Cologne

[3] Center for Molecular Medicine Cologne, University of Cologne

## Supplementary Methods

### Strains, media, and cultivation.

The recipient strain Bs166 (*his leu met amyE::$P_{hs}$comK(spc) comK::kan, $P_{comK}$gfp (CBL,cat)* (24) was derived from *B. subtilis* BD630. In this strain, the master regulator for competence, *comK*, is under control of an IPTG-inducible promoter. The reporter strain Bs175 (*his leu met amyE::PhscomK(spc) comK::kan lacA::PrrnE-gfp* (erm)) [1] carries an additional GFP reporter. The donor strains are *B. spizizenii* NRRL B-14472/W23 (hybrid library BSPIZ) and *B. vallismortis* DV1-F-3 (hybrid libraries BVAL, BVAL_single and evolved hybrid libraries).

*B. subtilis* strains were grown either in complex medium (CM) at 37 or 42 °C or in chemically defined medium supplemented with glucose (DM) or glycerol ($DM_{glycerol}$) at 37°C. CM and DM are based on the Spizizen salts (6 g/l $KH_2PO_4$, 14 g/l $K_2HPO_4$, 2 g/l $(NH_4)_2SO_4$, 1 g/l tri-sodium citrate dihydrate). CM was supplemented with 0.5 % D-glucose, 50 µg/ml L-histidine, L-leucine, and L-methionine, 0.02 % casamino acids, 0.1 % yeast extract, and 0.5 mg/ml $MgCl{\cdot}6H_2O$. DM was supplemented with 0.5 % glucose, 0.5 mg/ml sodium glutamate, 50 µg/ml L-histidine, L-leucine, and L-methionine, and 0.2 mg/ml $MgSO_4$. $DM_{glycerol}$ was based on the Spizizen salts without tri-sodium citrate dihydrate added and was supplemented with 0.2 mg/ml $MgSO_4$, 50 µg/ml L-histidine, L-leucine, and L-methionine, and 10% MEM-AA-solution (Roth) and 5% MEM-NEAA-solution (Roth). Supplementation with amino acids was necessary to support growth in this medium. Growth was monitored by measuring the $OD_{600}$ on an Infinite M200 plate reader (Tecan, Männedorf, Switzerland).

### Measurement of generation times under different growth conditions

For the four different growth conditions that combine 3 growth media and 2 temperatures (Table S2), we determined the generation time of the recipient strain Bs166 during exponential growth phase by analysing OD curves measured with a plate reader (Tecan, infinite M200 Pro).

Prior to the measurement, the cells were grown overnight in the medium and temperature in question. Then, cells were diluted into columns of a 96-well microtiter plate at 1:100, 1:1000, 1:10000 and growth curves were recorded every 6 minutes with 25 flashes of light with 600 nm wavelength for ~12 h until they reached stationary phase. Shaking amplitude was 2.5 mm and frequency was 250 rpm. The generation time $t_g$ was determined by measuring the time $t$ cells in the dilution series needed to make up for a 10 fold higher dilution and calculating $t_g = t \cdot$

$\frac{log(2)}{log(10)}$. For each condition, at least 60 growth curves were recorded on 3 individual days. Results are shown in Table S3.

**Generation of hybrid libraries BVAL and BSPIZ.**

In wild type *B. subtilis*, competence for transformation is transient, i.e. bacteria stochastically switch into the state of competence, and exit this state after ~ 2 h [2]. To mimic this behaviour, we used strain Bs166 [1] which carries the master regulator for competence, *comK*, under the control of an IPTG-inducible promoter and induced competence for 2 h in the presence of donor DNA (Fig. 1a).

To generate the hybrid libraries BVAL and BSPIZ, genomic DNA of the donor species was extracted using the Qiagen DNA Blood & Tissue kit. The predominating DNA fragment length specified for this kit is ~ 30 kbp. The recipient Bs166 was grown overnight at 37 °C on LB agar plates, a colony was picked, resuspended in CM, and then grown for 2.5 h at 37 °C in the liquid medium. For transformation, competence was induced with 600 µM IPTG and genomic DNA from *B. vallismortis* or *B. spizizenii*, respectively, at one genome equivalent per recipient cell. After 2 h, cells were washed thrice with phosphate-buffered saline (PBS) and diluted 1:100 in CM. We obtain a population of transformation hybrids from which we randomly pick hybrids to generate monoclonal libraries BVAL and BSPIZ.

To ensure monoclonality, we added two more steps to the protocol. Competent *B. subtilis* are growth-arrested and require ~ 2 h to resume growth after the escape from the competent state. To resolve heteroduplexes formed during transformation, one cell division is required [3]. Therefore, cells were grown until the OD doubled. Exponentially growing *B. subtilis* form chains. To ensure that chain formation does not interfere with monoclonality, 600 µM IPTG was added for 2 h to resolve the chains. During this procedure, cells grew for only few generations, and therefore, selection is minimal.

Then, cells were diluted 1:$10^5$, plated on LB agar, and grown over night at 37 °C. Next day, 88 single colonies were picked, grown in CM, mixed with DMSO (10% v/v), and stored at – 80 °C.

**Generation of BVAL_single library.** In the random replacement library BVAL, the orthologously replaced segments contain coding regions as well as regulatory regions of the genome. We addressed the question whether hybrids with individual donor genes replaced showed strong fitness effects. Genes for the BVAL_single library were randomly picked from

a list of *B. subtilis* genes having a homolog in *B. vallismortis.* Homologous genes were found using the Basic Local Alignment Search Tool (blast) by aligning *B. subtilis* genes to *B. vallismortis* and excluding those with 100 % sequence identity and all hits covering less than 90% of the gene's length [2].

When generating this library, we faced the challenge that the replacement had to be free of selective markers, as the latter might have introduced unwanted fitness effects. To this end, we adapted a method for generating marker-less (aka clean) deletion mutants [4] for generating clean replacements (Fig. S1)**.** Specifically, the construction of the BVAL_single library was performed by using the temperature-sensitive pMiniMad2 plasmid [4]. The method is based on single crossover integration of the plasmid into the chromosome at the target locus at 37°C in the presence of selection, where the vector replication is restricted and plasmid excision at the permissive temperature in the absence of selection.

Primers used for the generation of the BVAL_single library were designed by using the SnapGene® software (from Insightful Science) (Dataset S1). The gene replacement strategy is based on the following protocol. Regions of 500 bp up- (5'-UTR) and downstream (3'-UTR) of the target recipient gene and the donor gene were amplified by PCR under standard condition using Q5 polymerase (NEB). Purified DNA fragments containing overlapping parts (OP) were assembled with the NEBuilder Hifi DNA Assembly Master Mix (NEB) into the plasmid backbone, which was also amplified with the Q5 polymerase using the primers pMiniMad_fwd (5'-CACTGGCCGTCGTTTTAC-3') and pMiniMad_rev (5'-TGGCGTAATCATGGTCATAG-3'). The resulting plasmid was isolated from NEB10ß strain (NEB) and used to transform freshly prepared competent *B. subtilis* BD3836 [5]. After transformation cells were plated on LB agar plates supplemented with lincomycin (20µg/ml) and erythromycin (1µg/ml) and incubated overnight at 37°C. Eight single clones were randomly selected and grown in liquid LB medium with lincomycin and erythromycin at 37°C overnight. Next, transformants were cultured at room temperature for 2-3 days and diluted twice a day 1:30 in liquid LB medium. Several clones were tested via PCR for the gene replacement using *B. vallismortis* specific primers (Bval_spec), which bind only to the *B. vallismortis* gene. The loci of the positive clones were amplified by using primers seq_L and seq_R (Dataset S1) and sequenced by Eurofins (Ebersberg, Germany).

The BVAL_single library consists of 24 strains each having a different gene fully replaced by the donor's ortholog (Dataset S1) and additional 19 strains with a partial replacement.

**Generation of libraries of evolved hybrid populations.**

In the evolution assay, populations growing in parallel on 96-well microtiter plate were diluted regularly. For this, we used an automated system integrated by the company HighRes Biosolutions. The system consists of the following devices: Incubator (StoreX STX44, Liconic), liquid-handling device (Lynx LM900, Dynamics Devices), robotic arm (Acell, HighRes Biosolutions), plate reader (Synergy, BioTek Instruments), shaker (BioShake 3000 elm, QInstruments), plate storage (NanoServe, Acell, HighRes Biosolutions) and delidding device (LidValet, HighRes Biosolutions).

We started by performing one transformation step with *B. vallismortis* DNA, as explained for BVAL (Fig. 1a) resulting in a hybrid population. The population was washed, diluted to an OD of 0.0008 in either CM or DM and split up into 88 wells on a 96-well microtiter plate (~$1x10^5$ cfu/well). On this plate, hybrid populations were then grown in parallel at 37°C, at 500 rpm and 1 mm amplitude for ~450 generations, which amounted to 5 days in CM and 12.5 days in DM. The cells were continuously kept in exponential growth phase by individually diluting each well to OD 0.0008 after 4h in CM and 6h in DM, respectively. After ~ 450 generations, we plated each population on a LB agar plate and picked one hybrid per population, thus generating the monoclonal hybrid libraries BVALevoCM and BVALevoDM. As a reference for both libraries, 88 wells of the recipient Bs166 were evolved in the respective media and the libraries RECevoCM and RECevoDM were generated.

**High-throughput competition assay.**

For measuring the selection coefficients of different libraries under different growth conditions, and their respective controls, we developed high-throughput competition experiments by making use of the liquid-handling device (Lynx LM900, Dynamics Devices) and a flow-cytometer (Beckman Coulter) that reads out 96-well microtiter plates. To measure the relative fitness, strains are always competed against the reporter strain (RS), i.e. the *gfp*-expressing recipient Bs175.

To prepare for the competition experiment, all 88 strains of the library of interest were grown overnight in the medium later used for the competition on a 96-well microtiter plate together with 5 wells of the control strain (non-transformed Bs166), and 3 wells containing only medium. Additionally, RS was grown in an Erlenmeyer flask under the same conditions. For most competition experiments, we ensured that the bacteria were in exponential growth phase prior to the competition assay by diluting the overnight culture in medium and letting the cells

grow out of lag phase for 2 h and 6 h, in CM and DM, respectively. The only exception was the experiment in which we address the effect of the lag phase, where we immediately started the competition with the overnight culture (Table S2).

For the actual competition experiment, we diluted the cells in PBS to 0.01 OD and then mixed the cells on 96-well microtiter plates. We created 2-4 plates of mixes, aiming at a 1:1 ratio of competitors, by slightly varying the added volume of strain/RS cells. The actual starting fractions were determined with the flow-cytometer. All plates were diluted 1:10 in the growth medium of interest and grown for at least 14 generations in exponential growth phase (in CM at 37°C and 42°C: 4 h; in DM and $DM_{glycerol}$: 16 h) in a shaker at 37°C or 42°C, respectively. For the competition assay including lag phase, cells were grown for 6 h, ~ 2 h in lag and 4 h in exponential growth phase. Table S2 gives an overview of the different growth conditions. We prevented cells from reaching stationary phase. In CM, cells are still exponential after 4h. In the case of the 16h competition in DM and $DM_{glycerol}$, cells were diluted during competition to retain exponential growth. After the competition, all plates were diluted in PBS and the final ratio of strain/ RS cells was measured with the flow-cytometer.

The selection coefficient of the i-th strain was calculated with the fraction $x_i$ of the strain of interest and $x_{RS}$ of the reporter strain at the start $t_0$ and end time point $t$ as follows

$s_{i,RS} = \frac{t_g}{t-t_0} ln\left(\frac{x_i(t)/x_{RS}(t)}{x_i(t_0)/x_{RS}(t_0)}\right)$. $t_g$ is the generation time of the recipient in the respective media (Table S3). For the DFEs, the selection coefficients $s_{i,r}$ were calculated relative to the recipient's fitness measured on the same experimental plate (details in Supplementary Methods).

Each experiment ran independently on at least 3 days and the selection coefficients were averaged values over days and plates. Not all 88 library samples could be considered, as competitions with starting fractions of 40-60% measured on at least 2 days were required. Detailed information on the analysis of the flow-cytometry data is given below. For each library, the measured selection coefficients together represent the DFE for the applied conditions.

The control DFEs were determined for each growth condition for 82 samples of the recipient strain competing against RS with the same assay as used for the libraries.

**Analysis of flow cytometry data.** In the competition assay, we determined the fractions of the library strains $i$ and the competitor, the fluorescent reporter strain (RS), at the start and end time

point of competition. We measured about 30000 events per sample at the flow-cytometer, exported the raw data in the FCS3.0 format and processed it with Matlab after importing it with the fca_readfcs function [6]. To include all events ascribed to cells and exclude debris, we created a gate on the forward and side scatter data (FSC and SSC) and counted the events in the gates to obtain the raw fraction of strain $i$ and RS cells.

Two kinds of corrections were applied to the raw fractions determined by the flow-cytometer. First, during the competition experiment, three positions on the 96-well microtiter plate contained only RS cells that were used to correct for debris that may have been falsely detected as cells. We detected a fraction $d$ of events that was falsely categorized as non-fluorescent cells per plate but that in fact were due to impurities in the solution or non-fluorescing RS cells. As these events would have falsely been interpreted as non-*gfp*-expressing cells in the mixed samples, we applied the following correction. For each competition sample, we computed the fraction $d$ of measured RS and then increased the fraction of RS and decreased the fraction of strain $i$ by this amount. We note that $d$, amounted to a minor correction only. For the further analysis, we only considered samples with starting fractions of 40-60 %.

Second, we corrected for the slightly different fitness of the reporter strain RS compared to the recipient $r$ with the five wells on the 96-well plate that were used for recipient competition during every experiment. First, we measured for each strain $i$ the selection coefficient relative to the direct competitor RS in the same well as $s_{i,RS} = \frac{t_g}{t-t_0} ln\left(\frac{x_i(t)/x_{RS}(t)}{x_i(t_0)/x_{RS}(t_0)}\right)$. To calculate the coefficient $s_{i,r}$ relative to the recipient, we determined the mean selection coefficient of the recipient and RS as $\bar{s}_{r,RS}$ from the 5 recipient wells on the same plate with $s_{r,RS} = \frac{t_g}{t-t_0} ln\left(\frac{x_r(t)/x_{RS}(t)}{x_r(t_0)/x_{RS}(t_0)}\right)$. For each strain $i$, the selection coefficients were finally determined as $s_{i,r} = s_{i,RS} - \bar{s}_{r,RS}$ with the average of recipient fitness per day and plate. Afterwards, for each library strain, for every day the values from different plates were averaged and the results averaged over the individual measurement days. Each library was measured on at least 3 days and only samples were considered in the DFE that were successfully measured on at least 2 days.

**Statistical analysis of DFE.**

For each distribution of fitness effects, we defined the strains with large fitness effects as outliers. For this, we used the DFE of the control measurement obtained with the same assay and performed a two-sided z-test with each strain of the hybrid library, obtaining p-values. We

then applied the Bonferroni correction for multiple testing and defined outliers at the significance level of $\alpha = 0.05$. The DFE without the large effect transfer outliers was assumed to be dominated by the remaining small effect transfers.

In order to compare the distribution of small fitness effects for the different conditions, we first excluded outliers from the DFEs and then performed bootstrap analysis with 10000 resamples on the sample statistics mean and standard deviation. From the sampling distributions of the mean and standard deviation, we obtained the 95% confidence intervals for the sample statistics.

**Whole genome sequencing.**

Clonal genomes of ancestral and evolved populations were obtained using next generation sequencing (NGS) methods, in particular Illumina HiSeq. Samples were grown overnight in CM at 37 °C. A 2 ml aliquot of that culture was pelleted at 16.7 xg for 3 min, decanted, and then frozen at - 20 °C. Genomic DNA was isolated from the frozen pellet using the Qiagen DNeasy Blood & Tissue Kit (Hilden, Germany) according to the manufacturer's instructions. A small aliquot of the isolated DNA was run on a 1% agarose gel with a 1 kb plus DNA Ladder (Thermo Scientific) to check for degradation. Non-degraded samples were sent to Eurofins Genomics (Ebersberg, Germany) for NGS. Sequencing was performed on an Illumina HiSeq 3000/4000 system with 150 bp paired-end reads and an average depth of ~ 450. Sequence analysis was performed using the pipeline described in Power et al [1] and orthologous replacements, insertions, deletions and duplications as well as mutations were detected as described in the Supplementary Methods.

**Detection of orthologous replacement, insertion, deletion, and duplication.** We used the protocol developed by Power et al for detecting orthologously replaced segments [1]. In short, we created a master list of positions and bases representing point differences between donor and recipient genomes. The sequencing reads of the library strains were mapped to the recipient (NCBI RefSeq NC_000964.3, [7]) using Burrows-Wheeler Aligner (v.0.7.17) [8], and variants were called with the mpileup function from samtools (samtools 1.8) and the call function from bcftools (bcftools 1.8) [9]. At each donor-recipient divergence site, the sequence of the library strain has a recipient (R) or donor (D) consensus. We inferred transfer segments in the evolved sequence symmetrically in both directions, using the following algorithm: (1) The 3'-end of each segment is marked by a D site that is followed by a sequence of 5 consecutive R alleles.

The 3'-end coordinate of the segment was then assigned to the midpoint between the last D site and first of the five R sites. (2) The 5'-end of each transfer segment was marked by a D site that is preceded by a sequence of five consecutive R alleles. The 5'-end coordinate of the segment was then assigned to the midpoint between the last of the five R sites and the first D sites. Putting the 3'-ends and the 5'-ends together as orthologously replaced segment, only those were accepted that contain a minimum of two D sites.

Allele differences between library sequences and R/D consensus sequences that were not explained by orthologous replacement were ascribed to de novo point mutations. Alleles inserted or deleted that were not explained by orthologous replacement were ascribed to indels.

Start and end positions of the accessory recipient/donor genes were defined using the coverage of reads when mapping the sequenced donor reads on the recipient and vice versa. We calculated the coverage per base from the mapped reads using bedtools (v2.26.0) [10]. Parts of the genome, with a coverage below 50 for 150 subsequent bases we define to be the accessory genome. All genes within these regions are accessory genes. Every part of the genome that does not belong to the accessory genome has a homologue in the other species' genome and is called core genome.

To identify deletions and duplications the genome coverage, per base, of library strains mapped to recipient was calculated. After smoothing, the coverage with a sliding window of 30 bps, all segments in which the coverage dropped down to zero for at least ten bases in a row were defined to be deletions. These can be detected all over the genome of the library strains, both the core and the accessory regions. To be accepted as duplication, the coverage has to hit twice the genome-wide average coverage for ten subsequent bases.

We detected integrations of donor specific genes by mapping the sequencing reads of the library strains stringently to the donor. Without integrations from the donor specific genes, we would expect no coverage in the accessory regions of the donor. In case we found coverage in those regions, and it is a maximum of 1.5 $\sigma$ smaller than the genome-wide average coverage we assumed this part to be integrated into the library strain. Testing this method reveals a resolution down to 50 bp long integrations.

All protocols described above were tested using artificially designed sequencing data.

## Supplementary Figures

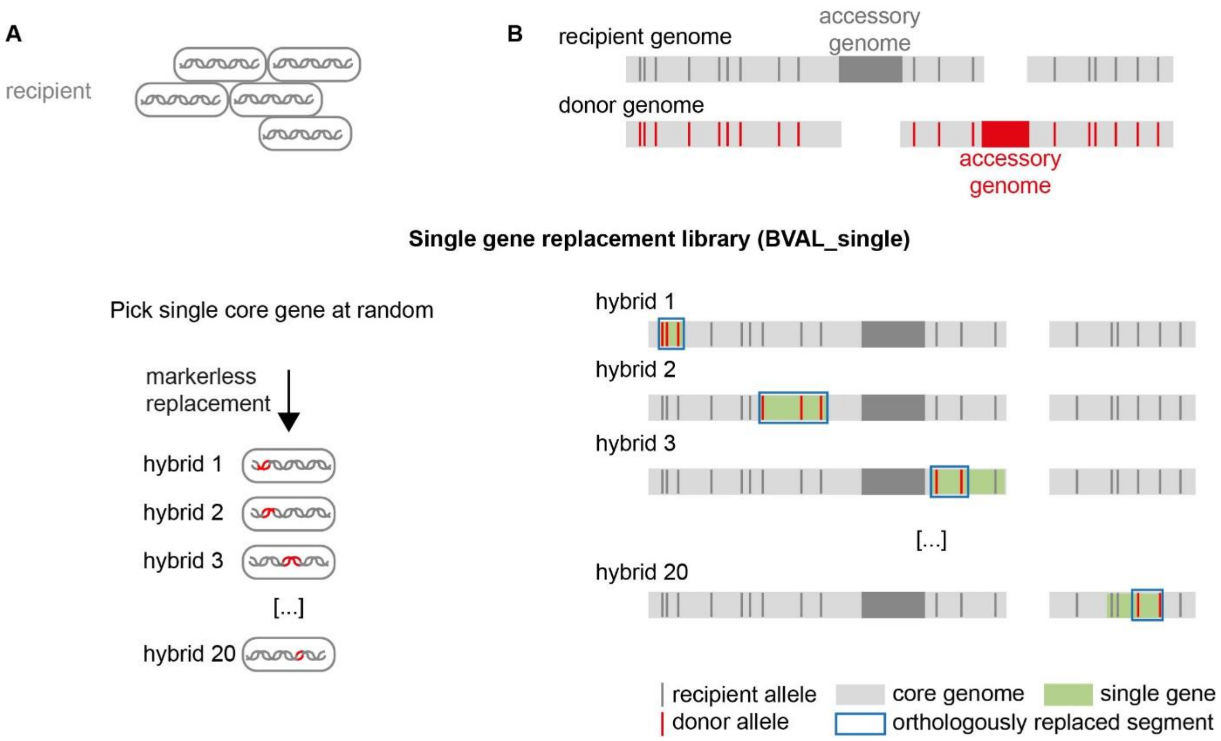


**Fig. S1 Generation of the single gene replacement library BVAL_single.** A) The single gene replacement libraries were generated by randomly selecting a core gene and fully or partially replacing the recipient allele by the donor allele by means of marker-less replacement. B) The library consists of hybrids in which a single core gene has been fully or partially replaced by its donor ortholog.

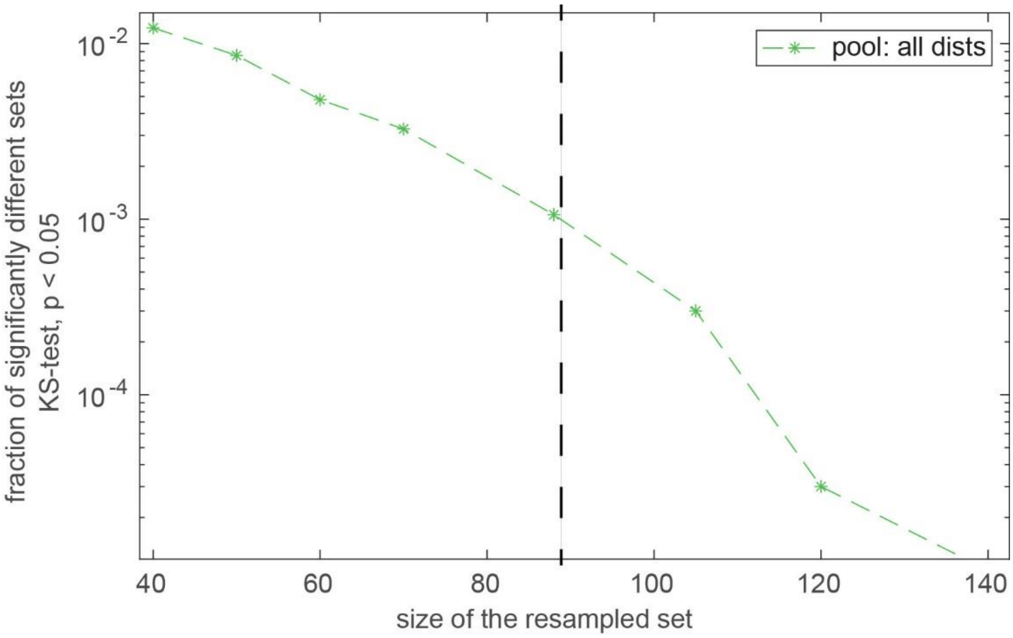


**Fig. S2 Subsampling analysis of DFE.** Random subsampling of the selection coefficients suggests that the experimentally determined DFEs are likely to be representative for the DFE of transformation. DFE data from the BVAL, BSPIZ and control measurement in complex medium are pooled and subsamples are randomly drawn from the DFE data 100000 times. Using a KS-test, each subsample is tested against the pooled data set and assigned a p-value. Fractions of resampled datasets that are significantly different from the pooled data set ($p < 0.05$) are shown as a function of sample size. We conclude that at a sample size of 88 strains, the probability that we do not describe the global shape of the "true" DFE correctly is on the order of $10^{-3}$.

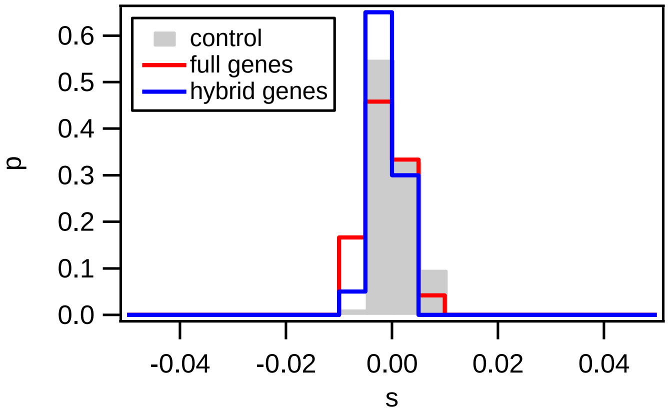


**Fig. S3 Analysis of BVAL_single library.** DFE form Fig. 2 whereby the DFEs of fully replaced genes and partially replaced genes are shown separately. Neither the mean nor the variance of the distributions is significantly different from the control distribution.

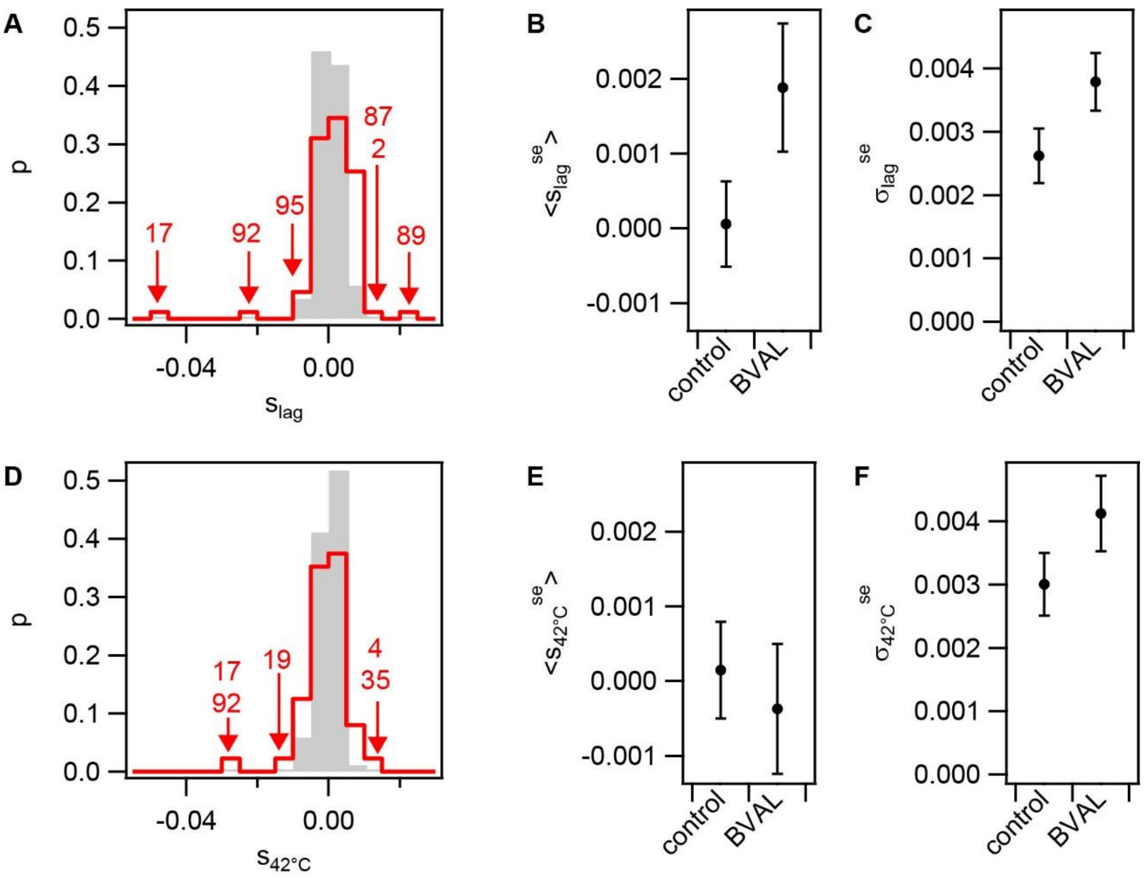


**Fig. S4 Distribution of fitness effects of BVAL library with lag phase and at 42 °C.** Selection coefficients of library BVAL were determined in complex medium including the lag phase A) - C) and at 42 °C D) – F). a) Distribution of selection coefficients $s_{lag}$ resulting from competition experiments between single strains of the BVAL library and the recipient expressing *gfp* (Bs175) are shown (red). Control distribution (grey). B) Mean selection coefficients $s_{lag}^{se}$ and C) standard deviation $\sigma_{lag}^{se}$ of core distributions after removing outliers. D) Distribution of selection coefficients $s_{42°C}$ resulting from competition experiments between single strains of the BVAL library and the recipient expressing *gfp* (Bs175) are shown (red). Control distribution (grey). E) Mean selection coefficients $s_{42°C}^{se}$ and F) standard deviation $\sigma_{42°C}^{se}$ of core distributions after removing outliers. Error bars: confidence intervals obtained from bootstrap analysis.

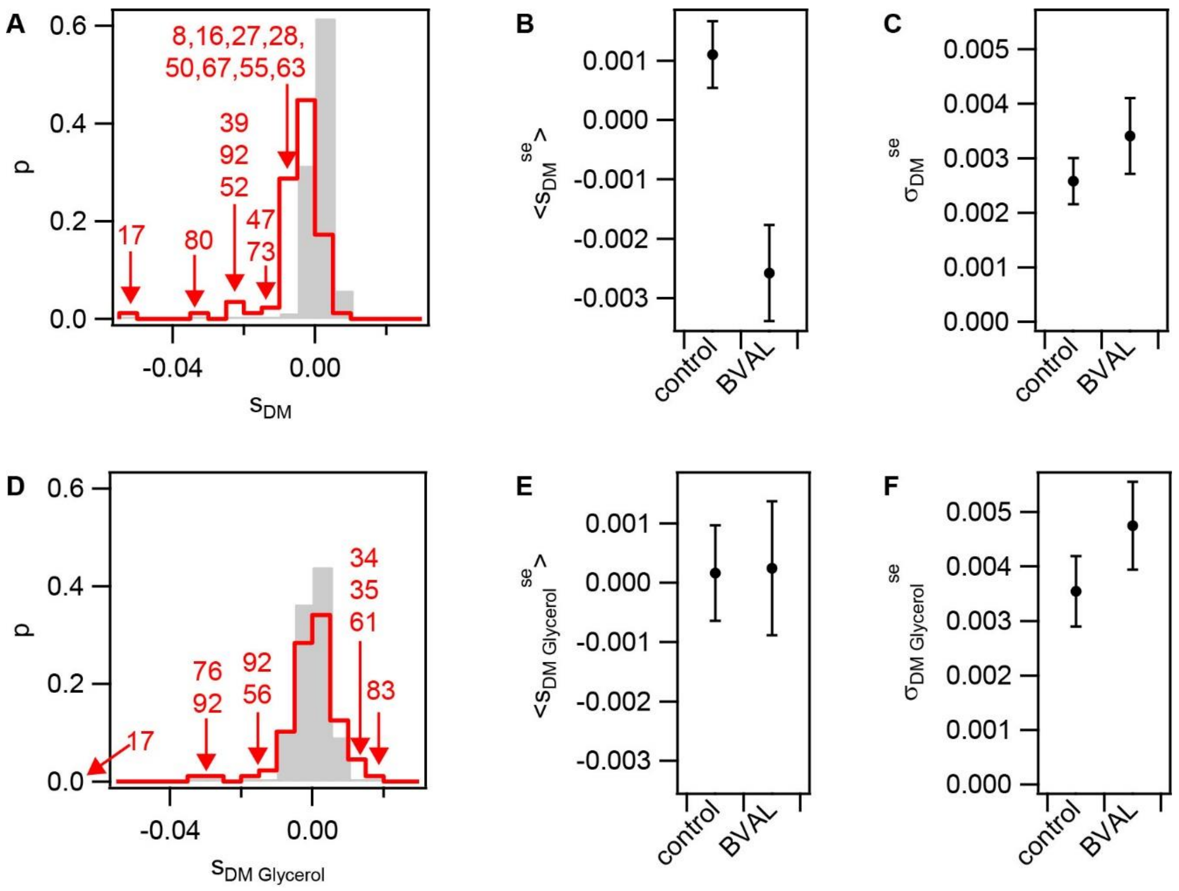


**Fig. S5 Distribution of fitness effects of BVAL library in defined medium.** Selection coefficients of library BVAL were were determined in complex medium. Competitors were mixed in exponential phase and grown for 16 h. A) Distribution of selection coefficients $s_{DM}$ resulting from competition experiments between single strains of the BVAL library and the recipient expressing *gfp* (Bs175) are shown (red). Control distribution (grey). B) Mean selection coefficients $s_{DM}^{se}$ and C) Standard deviation $\sigma_{DM}^{se}$ of core distributions after removing outliers. D) Distribution of selection coefficients $s_{DM\ Glycerol}$ resulting from competition experiments between single strains of the BVAL library and the recipient expressing *gfp* (Bs175) are shown (red). Control distribution (grey). E) Mean selection coefficients $s_{DM\ Glycerol}^{se}$ and F) Standard deviation $\sigma_{DM\ Glycerol}^{se}$ of core distributions after removing outliers. Error bars: confidence intervals obtained from bootstrap analysis.

## Supplementary Tables

| | *B. spizizenii (BSPIZ)* | *B. vallismortis (BVAL)* |
|---|---|---|
| # SNPs | 1274 (1977) | 334 (655) |
| fraction core genome replaced | 0.5 (0.8) % | 0.1 (0.2) % |
| length of replaced segment | 4080 (4598) | 1289 (1220) |
| # replaced segments | 4 (4) | 3 (4) |
| # hit genes | 21 (31) | 6 (11) |
| # insertions | 0.5 (0.5) | 0 |
| # deletions | 0.2 (0.2) | 0.1 (0.3) |
| # de novo mutations | 3 (8) | 0.3 (0.7) |

**Table S1 Genomic changes of ten randomly chosen strains from BSPIZ and BVAL**. A randomly chosen subset of ten strains of each library were sequenced. Mean (standard deviations) are shown.

| Medium | Competition in exp. phase | Temperature | Library (sample size) |
|---|---|---|---|
| CM | 4h | 37°C | BVAL (87), BVAL_single (43),<br>BSPIZ (87),<br>BVALevoCM (83), RECevoCM (86) |
| | 4h | 42°C | BVAL (88) |
| | 6h (incl. ~2h lag) | 37°C | BVAL (87) |
| DM | 16h | 37°C | BVAL (88)<br>BVALevoDM (88), RECevoDM (85) |
| DM_Glycerol | 16h | 37°C | BVAL (86) |

**Table S2: The hybrid libraries were examined under different growth conditions with the high-throughput competition assay.** Cells were mostly competed in exponential growth phase and the only exception was the measurement where the ~ 2h long lag phase was included (incl. ~ 2h lag). Library sizes can be less than 88, as selection coefficients could not be measured for all samples. Each library was characterized on at least 3 days and for each condition, a control was included with the same assay.

| Growth medium | Temperature [°C] | Generation time [min] |
|---|---|---|
| CM | 37 | 17,1 ±0,2 |
| CM | 42 | 14,9 ± 0,3 |
| DM | 37 | 39,0 ± 0,2 |
| $DM_{glycerol}$ | 37 | 48,3 ± 0,3 |

**Table S3 Generation time of the recipient strain Bs166 in different growth conditions.** Errors were calculated by performing error propagating with the standard error of the mean of each individual measurement.

**Dataset S1 Genes replaced in BVAL_single**

**Dataset S2 Genetic changes of 10 fittest evolved strains**

1. Power JJ, Pinheiro F, Pompei S, Kovacova V, Yuksel M, Rathmann I, et al. Adaptive evolution of hybrid bacteria by horizontal gene transfer. P Natl Acad Sci USA. 2021;118(10):e2007873118. doi: ARTN e200787311810.1073/pnas.2007873118. PubMed PMID: WOS:000627429100011.

2. Maier B. Competence and Transformation in Bacillus subtilis. Curr Issues Mol Biol. 2020;37:57-76. doi: 10.21775/cimb.037.057. PubMed PMID: WOS:000508654200005.

3. Dalia AB, Dalia TN. Spatiotemporal Analysis of DNA Integration during Natural Transformation Reveals a Mode of Nongenetic Inheritance in Bacteria. Cell. 2019;179(7):1499-511. doi: 10.1016/j.cell.2019.11.021. PubMed PMID: WOS:000502546200009.

4. Patrick JE, Kearns DB. MinJ (YvjD) is a topological determinant of cell division in Bacillus subtilis. Mol Microbiol. 2008;70(5):1166-79. doi: 10.1111/j.1365-2958.2008.06469.x. PubMed PMID: WOS:000261070300010.

5. Maamar H, Dubnau D. Bistability in the Bacillus subtilis K-state (competence) system requires a positive feedback loop. Mol Microbiol. 2005;56(3):615-24. doi: 10.1111/j.1365-2958.2005.04592.x. PubMed PMID: WOS:000228179100005.

6. fca_readfcs [Internet]. 2022.

7. O'Leary NA, Wright MW, Brister JR, Ciufo S, McVeigh DHR, Rajput B, et al. Reference sequence (RefSeq) database at NCBI: current status, taxonomic expansion, and functional annotation. Nucleic Acids Res. 2016;44(D1):D733-D45. doi: 10.1093/nar/gkv1189. PubMed PMID: WOS:000484575500002.

8. Li H, Durbin R. Fast and accurate short read alignment with Burrows-Wheeler transform. Bioinformatics. 2009;25(14):1754-60. doi: 10.1093/bioinformatics/btp324. PubMed PMID: WOS:000267665900006.

9. Danecek P, Bonfield JK, Liddle J, Marshall J, Ohan V, Pollard MO, et al. Twelve years of SAMtools and BCFtools. Gigascience. 2021;10(2):giab008. doi: ARTN giab00810.1093/gigascience/giab008. PubMed PMID: WOS:000637191300010.

10. Quinlan AR, Hall IM. BEDTools: a flexible suite of utilities for comparing genomic features. Bioinformatics. 2010;26(6):841-2. doi: 10.1093/bioinformatics/btq033. PubMed PMID: WOS:000275243500019.
